# Survival analysis and iniquities in older Brazilians: a six year follow up survey in São Paulo, Brazil

**DOI:** 10.1186/1475-9276-11-S1-A8

**Published:** 2012-01-23

**Authors:** Jair LF Santos, Maria Lúcia Lebrão, Yeda AO Duarte

**Affiliations:** 1 Department of Social Medicine, University of São Paulo, São Paulo, Brazil; 2 Department of Epidemiology, University of São Paulo, São Paulo, Brazil; 3 Department of Nursing Medical Surgery, University of São Paulo, São Paulo, Brazil

## Background

In Brazil, the aging process is fast with consequences for health services. By 2025 the elders will be more than 30 million people, 15% of Brazilian population. This study analyzes inequalities associated with survival of elders in a six year follow-up in Sao Paulo, Brazil.

## Methods

Data comes from a longitudinal survey – SABE Study (Health, Well Being and Aging) that began in 2000 with a sample of population aged 60+ living in São Paulo/Brazil (n=2,143 from a multi stage clustered sampling). A procedure with probability proportional to the size was carried out using census tracts with replacement. To achieve the desired oversampling for respondents aged 75+, additional households close to the selected census tracts were sampled. The second wave was done in 2006 when 1115 elders were re-interviewed. Descriptive statistics included tests for association using Rao Scott procedure with correction for sample-design. Multi variable analysis was done by adjusting Cox regressions with robust estimation, stratified by age and sex. Kaplan-Meier Survival Analysis was used to compare survival curves by social demographic conditions (sex, schooling, income, early conditions) and health (depression, comorbidities, disability, self-perceived health and falls).

## Results

Mortality rate was 55.2/1000years for males and 34.0/1000years for females. The demographic variables associated with survival, besides age and gender, were: greater education (p <0.0000), higher income (p <0.0000) and urban origin for women (p = 0.015). The health related variables were self-reported better health (p <0.000 for women and p = 0.016 for men), no self-reported disease (p <0.000), depression (p = 0.035 for women) and no disability (p <0.000). Cox regression showed clearly a gradient of increasing mortality with the decrease in income. In Kaplan-Meier analysis, absence of disability makes the male curve higher than the female.

## Conclusions

There are inequalities associated with lower survival. Public policies should take into account the needs of the elderly population to facilitate access to health care services and reduce inequities.

